# Identification of a diazinon-metabolizing glutathione *S*-transferase in the silkworm, *Bombyx mori*

**DOI:** 10.1038/srep30073

**Published:** 2016-07-21

**Authors:** Kohji Yamamoto, Naotaka Yamada

**Affiliations:** 1 Department of Bioscience and Biotechnology, Kyushu University Graduate School, Fukuoka, Japan

## Abstract

The glutathione *S*-transferase superfamily play key roles in the metabolism of numerous xenobiotics. We report herein the identification and characterization of a novel glutathione *S*-transferase in the silkworm, *Bombyx mori*. The enzyme (bmGSTu2) conjugates glutathione to 1-chloro-2,4-dinitrobenzene, as well as metabolizing diazinon, one of the organophosphate insecticides. Quantitative reverse transcription–polymerase chain reaction analysis of transcripts demonstrated that bmGSTu2 expression was induced 1.7-fold in a resistant strain of *B. mori*. Mutagenesis of putative amino acid residues in the glutathione-binding site revealed that Ile54, Glu66, Ser67, and Asn68 are crucial for enzymatic function. These results provide insights into the catalysis of glutathione conjugation in silkworm by bmGSTu2 and into the detoxification of organophosphate insecticides.

Glutathione (GSH) *S*-transferases (GSTs, EC 2.5.1.18) are cytosolic enzymes that are common in both prokaryotes and eukaryotes^1^. In mammals, seven GST classes (alpha, mu, pi, omega, sigma, theta, and zeta) have been recognized and can be differentiated on the basis of their amino acid sequences; sequence identity is about 50% shared within one class and less than 30% shared between the other classes^2^,^3^. There are six GST classes (delta, epsilon, omega, sigma, theta, and zeta) in dipteran insects, including *Anopheles gambiae*^4^ and *Drosophila melanogaster*^5^,^6^. GSH conjugation is indispensable for the detoxification of xenobiotics^7^,^8^. Insect GSTs can influence their sensitivity to insecticides^4^,^9^, and as the Lepidoptera are the main insect pests in agriculture, the study of lepidopteran GSTs is of great importance. We have so far characterized various GSTs (delta, epsilon, omega, sigma, theta, zeta, and an unclassified GST) in the silkworm, *Bombyx mori*, a lepidopteran model animal^10^,^11^,^12^,^13^,^14^,^15^,^16^; a sigma-class GST in the fall webworm *Hyphantria cunea*, one of the most serious lepidopteran pests of broad-leaved trees^13^; and a delta-class GST in *Nilaparvata lugens*, a rice crop pest^17^.

In the present paper, we describe the identification and classification of a novel GST isolated from *B. mori.* The substrate specificity of this GST helps reveal how insects deal with xenobiotic agents and contribute to a more detailed understanding of the GST system.

## Results

### Sequence of cDNA encoding bmGSTu2

**Figure 1:**
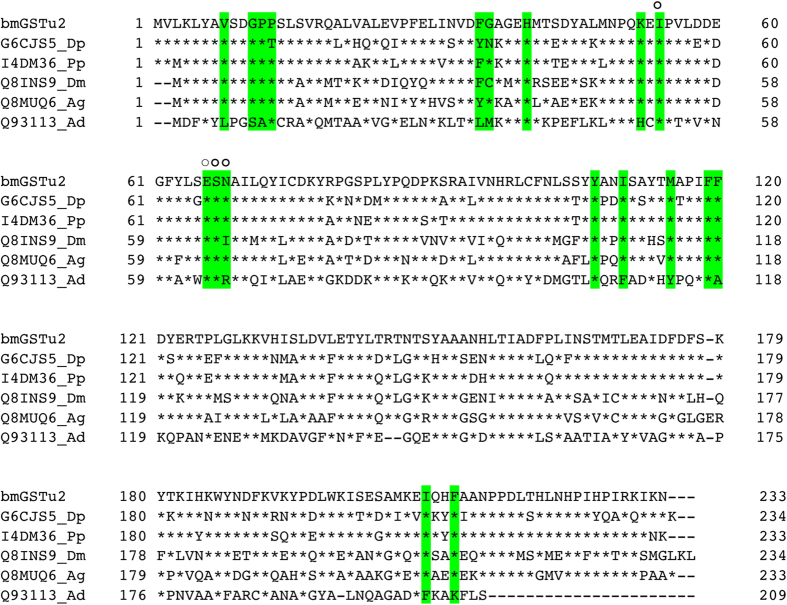
Amino acid sequences of unclassified glutathione *S*-transferases (GSTs). Sequences of GSTs were from the Swiss-Prot database (http://web.expasy.org/docs/swiss-prot_guideline.html). Aa, *Aedes aegypti*; Ag, *Anopheles gambiae*; Dm, *Drosophila melanogaster*; Dp, *Danaus plexippus*; Pp, *Papilio polytes*. The active site is shaded in green. Open circle indicates G-site of bmGSTu2. Asterisk represents identical amino acid and dash denotes a deletion.

**Figure 2:**
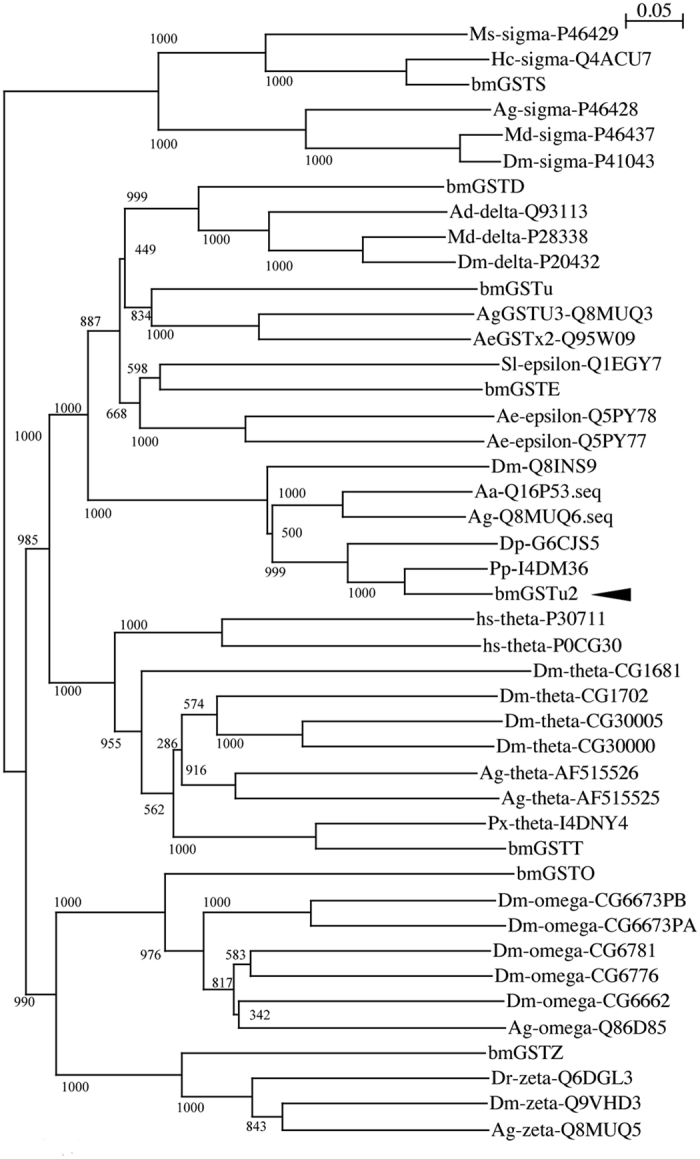
Phylogenetic tree of amino acid sequences of glutathione *S*-transferases (GSTs). The phylogenetic analysis was constructed by neighbor-joining plot software with GST sequences gained from the NCBI (http://www.ncbi.nlm.nih.gov/) and Swiss-Prot (http://web.expasy.org/docs/swiss-prot_guideline.html). Each entry includes the species name, GST class, and accession number. Branch length is represented by numbers, and numbers attached to nodes indicate bootstrap values. Ag, *Anopheles gambiae*; Md, *Musca domestica*, Dm, *Drosophila melanogaster*; Ms, *Manduca sexta*; Hc, *Hyphantria cunea*; Hs, *Homo sapiens*; Bm, *Bombyx mori*; Ae, *Aedes aegypti*; Sl, *Spodoptera litura*; Px, *Papilio xuthus*. The unclassified GST group does not contain a GST class. The arrowhead indicates bmGSTu2.

We obtained a cDNA, *bmgstu2*, from the fat body of silkworm and deposited the nucleotide sequence in GenBank under Accession No. LC054841. A BLAST search (http://blast.ncbi.nlm.nih.gov/Blast.cgi) in the Swiss-Prot database (http://web.expasy.org/docs/swiss-prot_guideline.html) revealed that the sequence corresponded to an unclassified GST. The sequence contained an open reading frame of 702 base pairs, encoding 233 amino acid residues (Fig. 1), and the amino acid sequence showed identities of 85%, 74%, 65%, 63%, and 32% to I4DM36_Pp, G6CJS5_Dp, Q16P53_Aa, Q8MUQ6_Ag, and Q93113_ad, respectively (Fig. 1). A phylogenetic tree showed that bmGSTu2 was clustered in the same clade with all other currently unclassified GST members (Fig. 2). Among *B. mori* GSTs, bmGSTu2 is closest to bmGSTE, bmGSTu, and bmGSTD, as shown in Fig. 2, and its amino acid sequence was 35% shared with that of bmGSTD, 31% shared with bmGSTu, and 30% shared with bmGSTE. The secondary structure of bmGSTu2 was predicted using SWISS-MODEL (http://swissmodel.expasy.org/workspace/). In addition, the STRIDE program^18^ showed that the bmGSTu2 monomer contains eight α-helices and four ß-strands. The locations of α-helices and ß-strands are conserved among the GSTs, including bmGSTD. The calculated molecular mass and isoelectric point (26,667 Da and 6.14, respectively) of bmGSTu2 are close to those of zeta- and delta-class GSTs from *B. mori*.

Previously, we measured the LD_50_ values for diazinon, a widely used organophosphate insecticide, in various strains of *B. mori*^15^. The most resistant and the most susceptible strains were named the R1 strain and S1 strain, respectively^15^. Expression of bmGSTu2 mRNA was investigated in the R1 strain using quantitative polymerase chain reaction (qPCR) analysis, with or without diazinon-treatment, using total RNA from fat bodies prepared as described above. The amount of amplified bmGSTu2 product from the fat bodies of the diazinon-treated larvae was about 1.7-fold that from the untreated controls.

### Tissue distribution of bmGSTu2 mRNA

**Figure 3:**
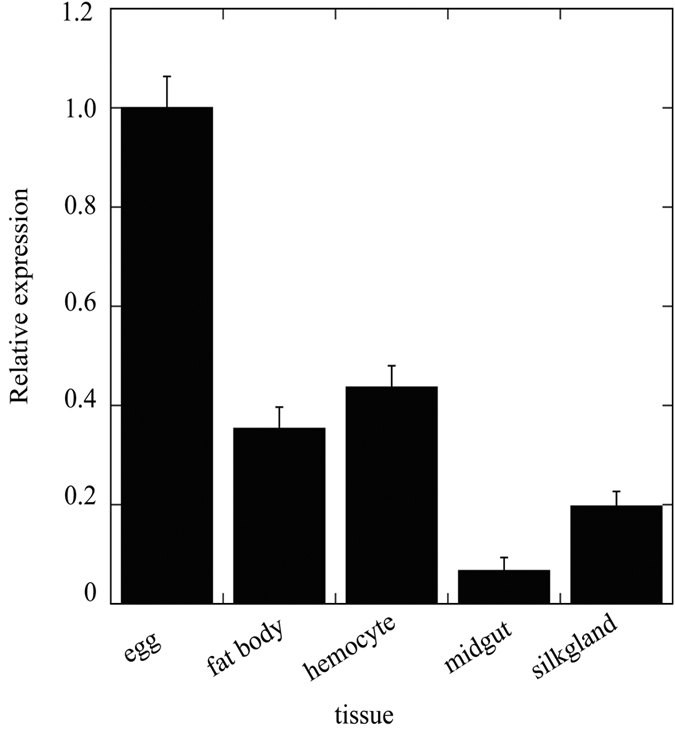
Localization of bmGSTu2 transcript. Quantitative polymerase chain reaction (qPCR) analysis was performed to detect bmGSTu2 transcripts in various tissues. Amounts of mRNA in various tissues were analyzed by qPCR as detailed in the Methods section. Data were normalized to *Bmrp49* mRNA levels. Error bars denote SD from three experiments.

Knowledge of the tissue distribution of bmGSTu2 mRNA expression can provide insight into its function. Figure 3 shows the presence of bmGSTu2 mRNA in adult tissues of silkworm that were examined by qPCR. The measurements used *Bmrp49* expression as an internal control. bmGSTu2 mRNA was detected in all tissues tested (Fig. 3), suggesting that bmGSTu2 is expressed ubiquitously. As endogenous substrates of bmGSTu2 are so far unknown, the function of bmGSTu2 in silkworm tissues remains obscure. Further understanding of the physiology of bmGSTu2 requires comprehensive study of the developmental changes of activity, protein, and mRNA in various tissues.

### Enzymatic properties of bmGSTu2

Bacterially produced bmGSTu2 was soluble and purified to near-homogeneity by affinity chromatography and gel-filtration (Supplementary Fig. S1). The final preparation yielded a single band on an SDS-PAGE gel with a molecular size of approximately 27,000 Da. This size is near the theoretical size by virtue of the amino acid sequence of bmGSTu2.

The characterization of purified bmGSTu2 was examined using 1-chloro-2,4-dinitrobenzene (CDNB) and GSH as substrates. bmGSTu2 was stable at temperatures <50 °C and maintained 75% of its original activity over a pH range of 6–9, narrower than that of other *B. mori* GSTs. The pH optimum of bmGSTu2 was 8.0, similar to the optimal pH levels of epsilon-, delta-, and sigma-class *B. mori* GSTs. Substrates other than CDNB were then used to profile the activity of bmGSTu2. Table 1 shows that bmGSTu2 demonstrated activity toward CDNB, 1,2-epoxy-3-(4-nitrophenoxy)-propane (EPNP), 4-nitrophenethyl bromide (4NPB), and ethacrynic acid (ECA), but it was inactive toward 4-nitrobenzyl chloride (4NBC), 4-hydroxynonenal (4HNE), or 4-nitrophenyl acetate (4NPA).

The activity of bmGSTu2 is lower than that of bmGSTT toward EPNP and 4NPB^19^. 4HNE is a favorite substrate for delta bmGST (bmGSTD) and omega bmGST (bmGSTO), while bmGSTu2 was relatively inactive against 4HNE. GSH peroxidase (GPx) activity was not detected in bmGSTu2, unlike in bmGSTO and epsilon bmGST. Kinetic parameters of bmGSTu2 toward CDNB were measured. The *K*_m_/*k*_cat_ value (34/min/mM), shown in Table 2, is higher than that of bmGSTE (3.35/min/mM), but lower than those of bmGSTD (268/min/mM) and bmGSTu (138/min/mM)^11^,^20^,^21^.

### Identification of metabolites

As GST is known to function as a detoxification enzyme, we examined whether bmGSTu2 could metabolize insecticides. Subsequent high performance liquid chromatography (HPLC) analysis revealed that bmGSTu2 was able to recognize diazinon as a substrate among 4,4′-diichlorodiphenyltrichloroethane (DDT), permethrin, diazinon, imidacloprid, and bendiocarb (Table 1). Optimal conditions for diazinon metabolism by bmGSTu2 were 30 °C and pH 7. Supplementary Fig. S2 shows that bmGSTu2 produced a new metabolite. MS analysis of the peak at 4.2 min showed the molecule ion *m*/z 442.1680[M+H^+^] (calculated C_18_H_28_N_5_O_6_S^+^, 442.1683) (Supplementary Figs S2 and S3). The precursor ion (*m*/z 442.1680) was fragmented by collision-induced dissociation to produce product ion masses of *m/z* at 313.1385 [M+H^+^-129.0295] by loss of γ-glutamyl moiety, and 169.0818 [M+H^+^-273.0862] by loss of γ-glutamyl-alanyl-glycine moiety (Supplementary Fig. S3). The molecule ion and the fragmentation pattern indicate that the metabolite is *S*-(2-isopropyl-4-methyl-6-pyrimidinyl glutathione. The diazinon and GSH conjugation caused by bmGSTu2 were involved in a substitution of the phosphothioester moiety of diazinon by GSH. In the case of the CDNB metabolite, the chlorine atom in CDNB was replaced by GSH (Supplementary Figs S4 and S5).

### Amino acid residues involved in enzymatic function

**Figure 4:**
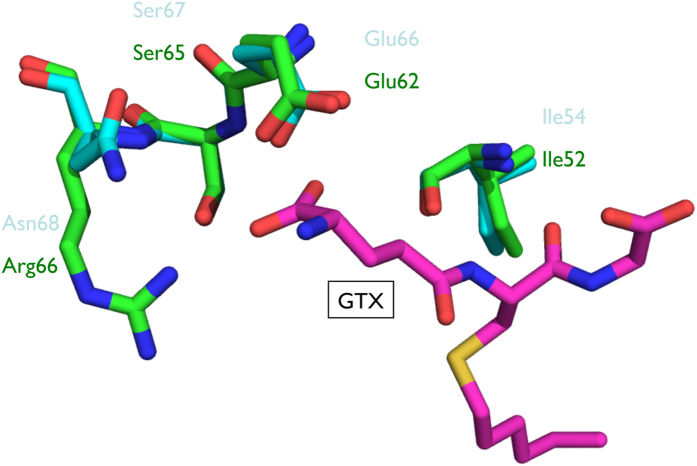
Superimposed structures of bmGSTu2 with agGSTd1-6 displaying amino acid residues of the G-site. Carbon atoms for bmGSTu2, agGSTd1-6, and GTX are cyan, green, and magenta, respectively. Other atoms of oxygen, nitrogen, and sulfur are represented by red, blue, and yellow, respectively. Names of amino acid residues for bmGSTu2 and agGSTd1-6 are shown in cyan and green, respectively.

The active site of GST has two components, the G-site (where GSH binds) and the H-site (where the hydrophobic substrate binds). The diversity of amino acids at both GST binding sites is associated with substrate selectivity. The G-site identified in bmGSTD includes Ser11, Gln51, His52, Val54, Glu66, Ser67, and Arg68^20^. Superimposition of modeled bmGSTu2 on bmGSTD suggests that equivalent residues include Gly11, Gln51, Lys52, Ile54, Glu66, Ser67, and Asn68 (data not shown). Since this model does not include GSH or its analogue, another superimposition of modeled bmGSTu2 was carried out on the delta-class GST of *A. gambiae* (agGSTd1-6, PDB code: 1PN9) (Fig. 4). The active site of agGSTd1-6 contains Leu6, Ser9, Ala10, Pro11, Lue33, Met34, His38, His50, Ile52, Glu64, Ser65, Arg66, Tyr105, Phe108, Tyr113, Ile116, Phe117, Phe203, and Phe207. Corresponding residues in bmGSTu2 include Val8, Gly11, Pro12, Pro13, Phe35, Gly36, His40, Lys52, Ile54, Glu66, Ser67, Asn68, Tyr107, Ile110, Met115, Phe119, Phe120, Ile208, and Phe211 (Fig. 1). The structural data suggest that four residues (Ile54, Glu64, Ser67, and Asn68) in bmGSTu2 could interact with *S*-hexylglutathione (GTX), an analogue of GSH.

Using the G-site of bmGSTD, we identified Ile54, Glu66, Ser67, and Asn68 as the candidate G-site of bmGSTu2 (Figs 1 and 3). To learn whether these residues are crucial for bmGSTu2 activity, site-directed mutagenesis was carried out. The resulting mutants were named I54A, E66A, S67A, and N68A and were prepared from *E. coli* clones (Supplementary Fig. S1). The kinetic parameters of the bmGSTu2 mutants were compared with those of the wild-type enzyme using CDNB and GSH as substrates (Table 2). The catalytic efficiencies of mutants toward CDNB and GSH were decreased, compared with that of the wild-type enzyme. The most prominent change in *k*_cat_/*K*_m_ was observed in the E66A mutant. As the activity of bmGSTu2 toward diazinon did not fit the Michaelis-Menten equation, we measured the conjugation activity of diazinon by the mutants. The activities of all mutants were insignificant. These results suggest that interactions between GSH and Ile54, Glu66, Ser67, and Asn68 of bmGSTu2 are crucial for the activity.

## Discussion

Regarding the GSTs that have been identified in *B. mori*, little is known about the unclassified GSTs. In the silkworm genome sequence (http://sgp.dna.affrc.go.jp/KAIKObase/), we found 23 homologs of GSTs, including unclassified (2 isoforms) GSTs. By comparison, the *A. gambiae* genome includes 3 unclassified GSTs, and *D. melanogaster* and *Apis mellifera* include no unclassified GSTs. The present study focused on the molecular and biochemical properties of a silkworm unclassified GST that had not been thoroughly investigated.

Previously, we screened diazinon-resistant and susceptible strains of silkworms^15^. bmGSTu2 mRNA was induced 1.7-fold in the resistant strain, whereas bmGSTu mRNA was induced by a factor of 2.5. Both unclassified GSTs are upregulated in the diazinon-resistant silkworm after diazinon exposure. Similar results have been reported in other insects: GST activity increased in a brown planthopper that was resistant to insecticide^22^ and in an abamectin-resistant vegetable leafminer^23^. Thus, the increase in GST expression could result in resistance to insecticide by enhancing the insect’s ability to metabolize the insecticide.

We focused on the specific activity of bmGSTu2 to examine whether GST activity contributed to the detoxification of diazinon, as well as the universal substrate of GSTs. bmGSTu2 catalyzed a broad range of reactions, compared with other GSTs of *B. mori* (Table 1). bmGSTu2 was not reactive with 4HNE, a cytosolic product of lipid peroxidation^24^, or to H_2_O_2_ as the substrate. We propose that the enzyme could not participate in the response to oxidative stress. HPLC analyses showed that bmGSTu2 was able to conjugate diazinon on GSH, in contrast with the results with other *B. mori* GSTs. Our results indicated that bmGSTu2 is a diazinon-metabolizing enzyme, suggesting that it may detoxify diazinon in the silkworm. This finding is consistent with other reports that the epsilon-class GSTs of mosquitoes likely confer their resistance to insecticides^25^,^26^.

It is accepted that the amino acid sequence of GST is separated into two regions, the N- and C-terminal domains^27^. The N-terminal domain includes the G-site, and the C-terminal domain has a substrate-binding site (H-site). The sequence variety of the H-site is responsible for substrate specificity^27^; furthermore, this variety may explain the varied substrate specificities of *B. mori* GSTs, since we found considerable differences between their C-terminal regions (alignments not shown). In contrast with the H-site, amino acid residues in the G-site are conserved in *B. mori* GSTs^11^,^19^,^20^,^21^,^28^,^29^. The mutagenesis experiments suggest that the residues Ile54, Glu66, Ser67, and Asn68 in bmGSTu2 play crucial roles in its enzymatic functions. In almost all cases, site-directed mutagenesis experiments revealed that the equivalent residues for Glu66 and Ser67 in bmGSTu are critical for the catalysis. In the epsilon-, omega-, and unclassified classes of *B. mori* GSTs, mutation of the equivalent residues for Ile54 to Ala affected enzyme catalysis^11^,^21^,^29^. Asn68 of bmGSTu2 also plays an important role in the catalytic reaction. Similar notices were reported for a delta-class GST of *B. mori* (bmGSTD)^20^, in which mutation of the equivalent residue (Arg68) of bmGSTD affected the enzymatic activity.

As described, the diversity of amino acids at the H-site of GST is associated with substrate selectivity. To determine how a suitable substrate may bind to the enzyme, a Dali search was performed to identify enzymes bearing the highest structural homology to bmGSTu2. Members of delta-class GSTs consistently ranked the highest, with root mean square deviations of 0.6–1.3 Å. Among the GSTs, the H-site of delta-class GSTs of *A. gambiae* (adGSTd1-6) was the most similar. The H-site of agGSTd1-6 (PDB ID: 1PN9) contains residues that are mostly hydrophobic: Leu6, Ala10, Pro11, Leu33, Met34, Tyr105, Phe108, Tyr113, Ile116, Phe117, Phe203, and Phe207^30^. In the sequence of bmGSTu2, five of the 12 residues (Pro13, Tyr107, Ile118, Phe119, and Phe211) are identical to those in agGSTd1-6, and the remainder (Val8, Phe35, and Ile110) are similar to the above agGST1-6 residues. We are pursuing co-crystallization of bmGSTu2 with a suitable insecticide–GSH conjugate to gain further understanding of the molecular basis for detoxification of xenobiotics by this novel GST.

Other amino acid residues may be necessary for bmGSTu2 catalytic activity, in addition to Ile54, Glu66, Ser67, and Asn68. GSH residues that are important for activity in the active site of the N-terminal domain of GST have been reported^4^,^5^. An electron-sharing network and lock-and-key motif are necessary for catalytic activity of GSTs^31^,^32^,^33^,^34^. Investigation of putative catalytic residues using site-directed mutagenesis is currently in progress in our laboratories.

The results suggest that bmGSTu2 could play a part in detoxification of diazinon in *B. mori*. Together with bmGSTu2, the functions of other GSTs in *B. mori* should be further investigated in order to know the defense reactions underlying insecticide. Future research will assist in the design and execution of strategies to handle insecticide-resistance in agricultural pests.

## Methods

### Insects and tissue dissection

Larvae of the silkworm (*B. mori*) were reared on mulberry leaves at the Institute of Genetic Resources, Kyushu University Graduate School (Fukuoka, Japan). Each tissue was dissected on ice from day-3 fifth-instar larvae and stored at −80 °C until use. Total RNA was prepared from the isolated tissues using an RNeasy Plus Mini Kit (Qiagen, Valencia, CA, USA), following the manufacturer’s protocol, and then used for RT-PCR.

### Cloning and sequencing of cDNA encoding bmGSTu2

The cDNA encoding bmGSTu2 was cloned from total RNA using RT-PCR. First-strand cDNA was synthesized using SuperScript II reverse transcriptase (ThermoFisher Scientific, Carlsbad, CA) and an oligo-dT primer. The cDNA obtained was used as a PCR template with the oligonucleotide primers bmGSTu2aF 5-CCGGAATTCATGGTTCTAAAATTATATGCCG-3′ (sense) and bmGSTu2aR 5-CCGCTCGAGATTTTTGATTTTTCGGATAGGG-3′ (antisense), using a sequence found from the SilkBase EST database^35^. *Eco*RI and *Xho*I restriction enzyme sites were represented by underlined and double-underlined regions in the primer sequences, respectively. PCR used the following protocol: 94 °C for 2 min, then 35 cycles of 94 °C for 1 min, 59 °C for 1 min, and 72 °C for 2 min; followed by 72 °C for 10 min. The resulting bmGSTu2 cDNA (*bmgstu2*) was ligated into the pGEM-T Easy Vector (Promega, Madison, WI), which was then transformed to *E. coli* DH5α cells. Genetyx software (ver. 18.0.4, Genetyx Corp., Tokyo, Japan) was used to obtain the complete sequence of *bmgstu2* and to deduce its corresponding amino acid sequence. Homology alignment (Fig. 1) was performed using Genetyx software (ver. 18.0.4), with the gap penalty (Insert: -12, Extend: -4). A phylogenetic tree was generated using neighbor-joining plot software (http://www-igbmc.u-strasbg.fr/Bioinfo/ClustulX/Top.html).

### Quantitative PCR (qPCR) analysis

cDNAs were prepared as described. qPCR Primer sets for bmGSTu2 and *B. mori* ribosomal protein 49 (Bmrp49) were designed. The primer sequences were as follows:

bmGSTu2bF (forward) 5-AGCCTACACAATGGCACCTATATTC-3′

bmGSTu2bR, (reverse) 5-CGCCGCATAGGATGTGTTT-3′

Bmrp49F, (forward) 5-GATGTGTTTTATATTC-3′

Bmrp49R, (reverse) 5-GCATCATCAAGATTTCCAGCTC-3′.

qPCR was performed on a Dice Real TimeSystemTP-800 Thermal Cycler (Takara) using SYBR Premix Ex Taq™ (Takara). PCR amplification began with a 10-s denaturation step at 95 °C and then 40 cycles of denaturation at 95 °C for 5 s, annealing at 55 °C for 20 s, and extension at 72 °C for 20 s. The samples were analyzed in triplicate, and bmGSTu2 levels were normalized against corresponding Bmrp49 levels and expressed as the bmGSTu2/Bmrp49 ratio.

### Overproduction and purification of recombinant protein

The *bmgstu2* insert was removed by digestion with *Eco*RI and *Xho*I and subcloned into the expression vector pET-22b, which was then used to transform competent *E. coli* Rosetta (DE3) pLysS cells (Novagen, EMD Biosciences, Inc., Darmstadt, Germany). The *E. coli* cells were then grown at 37 °C in Luria-Bertani media including 100 μg/mL ampicillin. After cell density (OD_600_) reached 0.8, isopropyl-1-thio-ß-D-galactoside was supplied at a final concentration of 1 mM to overexpress recombinant protein. The culture was incubated for additional 3 h, and cells were collected by centrifugation. The harvested cells were resuspended in 20 mM Tris-HCl buffer (pH 8.0) including 0.5 M NaCl, 4 mg/mL lysozyme, and 1 mM phenylmethanesulfonyl fluoride, and were sonicated. Unless otherwise noticed, purification steps stated below were conducted at 4 °C. The supernatant, including the recombinant protein, was obtained by centrifugation at 10,000 × *g* for 15 min and applied to Ni^2+^-affinity chromatography equilibrated with 20 mM Tris-HCl buffer (pH 8.0) containing 0.2 M NaCl. Samples were washed with the same buffer and eluted with a linear gradient of 0–0.5 M imidazole. Fractions containing the enzyme, assayed as described below, were pooled, concentrated using a centrifugal filter (Millipore Corp., Billerica, MA), and subjected to a Superdex 200 column (GE Healthcare Bio-Sciences, Buckinghamshire, UK) equilibrated with the same buffer, but with the addition of 0.2 M NaCl. Each fraction was assayed and was loaded to SDS-PAGE using a 15% polyacrylamide slab gel containing 0.1% SDS^36^. Protein bands were visualized by Coomassie Brilliant Blue R250 staining. Protein concentrations were assessed using a Protein Assay Kit (Bio-Rad Laboratories, Inc., Hercules, CA, USA), with bovine serum albumin as a standard.

### Molecular modeling

A structural model of bmGSTu2 was prepared by SWISS-MODEL (http://swissmodel.expasy.org)^37^ using the amino acid sequence. The model revealed a GMQE (Global Model Quality Estimation) score of 0.64^38^. The preparation of the bmGSTu2 model used the structure of bmGSTD (PDB code: 3VK9). The secondary structure assignments were produced with STRIDE^18^. A Dali search (http://ekhidna.biocenter.helsinki.fi/dali_server/) revealed structural homology between bmGSTu2 and bmGSTD with a root-mean-square deviation of 0.60 Å/214 residues for all atoms. The amino acid sequence of bmGSTu2 showed 35% shared identity with bmGSTD. Figures were drawn using Coot^39^ and PyMOL (http://pymol.sourceforge.net).

### Site-directed mutagenesis

Alanine substitution mutants of bmGSTu2 were constructed using the Quick-Change Site-Directed Mutagenesis Kit (Stratagene Corp., La Jolla, CA), based on the manufacturer’s instructions. An expression plasmid harboring *bmgstu2* was used as a template, and full-length mutated cDNAs were checked by DNA sequencing.

### Measurements of enzyme activity

GST activity was measured spectrophotometrically using 1-chloro-2,4-dinitrobenzene (CDNB) and 5 mM GSH as standard substrates^40^. Enzymatic activity was shown as mol CDNB conjugated with GSH per min per mg of protein. Kinetic parameters (*K*_m_ and *k*_cat_) were determined by a nonlinear least-squares data fit under assay conditions with various substrate concentrations in the presence of 5 mM GSH. Thermostability of bmGSTu2 was assessed by pre-incubation of enzyme solutions at different temperatures for 30 min prior to a residual activity assay. The pH stability of bmGSTu2 was examined by pre-incubation of enzyme solutions at various pH values at 4 °C for 24 h prior to a residual activity assay. Optimal pH for bmGSTu2 activity was assessed using citrate-phosphate-borate buffer at various pH values with a fixed ionic strength of 0.25.

### HPLC and mass spectrometry (MS)

The ability of bmGSTu2 to metabolize each insecticide was determined by HPLC conducted according to previous reports^11^,^17^,^19^. Briefly, reaction mixtures (500 μL) contained 10 mM diazinon, 5 mM GSH, and 20 μg bmGSTu2 in citrate-phosphate-borate buffer (pH 7, ionic strength of 0.25) and were incubated at 30 °C for 1 h. The mixtures were extracted with 500 μL of methanol for HPLC analysis. The eluate was monitored at 246 nm for the detection of metabolites. The concentration of each insecticide was determined on the basis of the corresponding peak area identified by its authentic sample.

The metabolites produced by bmGSTu2 were identified using an LCMS-IT-TOF mass spectrometer (Shimadzu Inc., Kyoto, Japan). The reacted sample (5 μL) described above was applied to reverse-phase HPLC on a CAPCELL PAK C18 column (150 × 3.0 mm i.d.; Shiseido Fine Chemicals, Tokyo, Japan), with solvent (A): 0.1% (v/v) formic acid/H_2_O, and solvent (B): 0.1% (v/v) formic acid/methanol, at 40 °C, with a flow rate of 0.20 mL/min. The metabolite was eluted on a linear solvent gradient program using the following steps: 50% solvent (B) for 2 min, 50–100% solvent (B) for 5 min, and 100% solvent (B) for 13 min for diazinon metabolite analysis, and 30% solvent (B) for 2 min, 30–100% solvent (B) for 7 min, and 100% solvent (B) for 10 min for CDNB metabolite analysis. The MS was operated with probe voltage of 1.70 kV, collision-induced dissociation temperature at 200 °C, block heater temperature at 200 °C, nebulizer gas (nitrogen) flow at 1.5 L/min, ion accumulation time for 50 ms, tolerance 0.05 *m/z*, MS range of *m/z* from 100 to 1000, and MS/MS range of *m/z* from 100 to 1000. The IT-TOF mass spectrometer was operated in the data-dependent acquisition mode using LCMS-solution version 3.50 software (Shimadzu).

## Additional Information

**How to cite this article**: Yamamoto, K. and Yamada, N. Identification of a diazinon-metabolizing glutathione *S*-transferase in the silkworm, *Bombyx mori.* *Sci. Rep.* **6**, 30073; doi: 10.1038/srep30073 (2016).

## Supplementary Material

Supplementary Information

## Figures and Tables

**Table 1:** Substrate selectivity of bmGSTu2.

Substrate	Concentration (mM)	Activity (μmol/min/mg)	Wavelength (nm)	Δε(mM^−1^cm^−1^)
CDNB	1.0	0.19 (0.038)	340	9.6
EPNP	1.0	0.79 (0.13)	260	0.5
4NBC	1.0	0.068 (0.017)	310	1.9
4NPB	1.0	0.55 (0.11)	310	1.2
4HNE	0.1	NA	224	13.8
ECA	1.0	0.29 (0.052)	270	5.0
4NPA	1.0	NA	400	8.3
H_2_O_2_	0.2	NA	340	−6.2
permethrin	0.25	NA	—	—
bendiocarb	0.25	NA	—	—
imidacloprid	0.25	NA	—	—
DDT	0.1	NA	—	—
diazinon	0.25	detected	—	—

Assay was performed at pH 8 in the presence of 5 mM GSH. Data are represented as mean (SD) of three independent experiments. NA represents no activity. Wavelength and Δε indicate maximum wavelength of the absorption and molecular coefficient, respectively. —: not applicable. CDNB, 1-chloro-2,4-dinitrobenzene; EPNP, 1,2-epoxy-3-(4-nitrophenoxy)-propane; 4NBC, 4-nitrobenzyl chloride; 4NPB, 4-nitrophenethyl bromide, 4HNE, 4-hydroxynonenal; ECA, ethacrynic acid; 4NPA, 4-nitrophenyl acetate; DDT, 4,4′-diichlorodiphenyltrichloroethane.

**Table 2:** Comparison of kinetic data for bmGSTu2 in complex with 1-chloro-2,4-dinitrobenzene (CDNB) and glutathione (GSH).

	Wild-type	I54A	E66A	S67A	N68A
CDNB					
* K*_m_	0.27 (0.082)	2.5 (0.83)	0.91 (0.26)	1.6 (0.31)	0.53 (0.17)
* k*_cat_	9.3 (2.2)	4.2 (0.88)	2.9 (0.31)	5.1 (0.75)	5.1 (1.4)
* k*_cat_/*K*_m_	34	4.2	3.2	3.2	9.6
GSH					
* K*_m_	0.91 (0.18)	2.9 (0.63)	6.9 (1.1)	0.88 (0.27)	5.6 (1.2)
* k*_cat_	5.0 (0.96)	3.4 (0.33)	4.3 (1.2)	1.64 (0.42)	3.6 (0.93)
* k*_cat_/*K*_m_	5.5	1.2	0.62	1.9	0.64

The values of *K*_m_ and *k*_cat_ are expressed as the mean (SD) of three independent experiments. *K*_m_, *k*_cat_, and *k*_cat_/*K*_m_ are expressed as mM, min^−1^, and M^−1^ min^−1^, respectively.
