# Supplementary material for: Identification of a diazinon-metabolizing glutathione S-transferase in the silkworm, Bombyx mori
Source: Sci Rep. 2016 Jul 21;6:30073. doi: 10.1038/srep30073 (PMC4954967; doi:10.1038/srep30073)
Supplement: Supplementary Information [file srep30073-s1.pdf]

Identification of a diazinon-metabolizing glutathione *S*-transferase in the silkworm, *Bombyx mori*

Kohji Yamamoto*, Naotaka Yamada

Department of Bioscience and Biotechnology, Kyushu University Graduate School, Fukuoka, Japan

*Corresponding author:

Kohji Yamamoto

6-10-1 Hakozaki, Higashi-ku, Fukuoka 812-8581, Japan

Tel.: +81-92-621-4991; Fax: +81-92-624-1011;

E-mail: yamamok@agr.kyushu-u.ac.jp

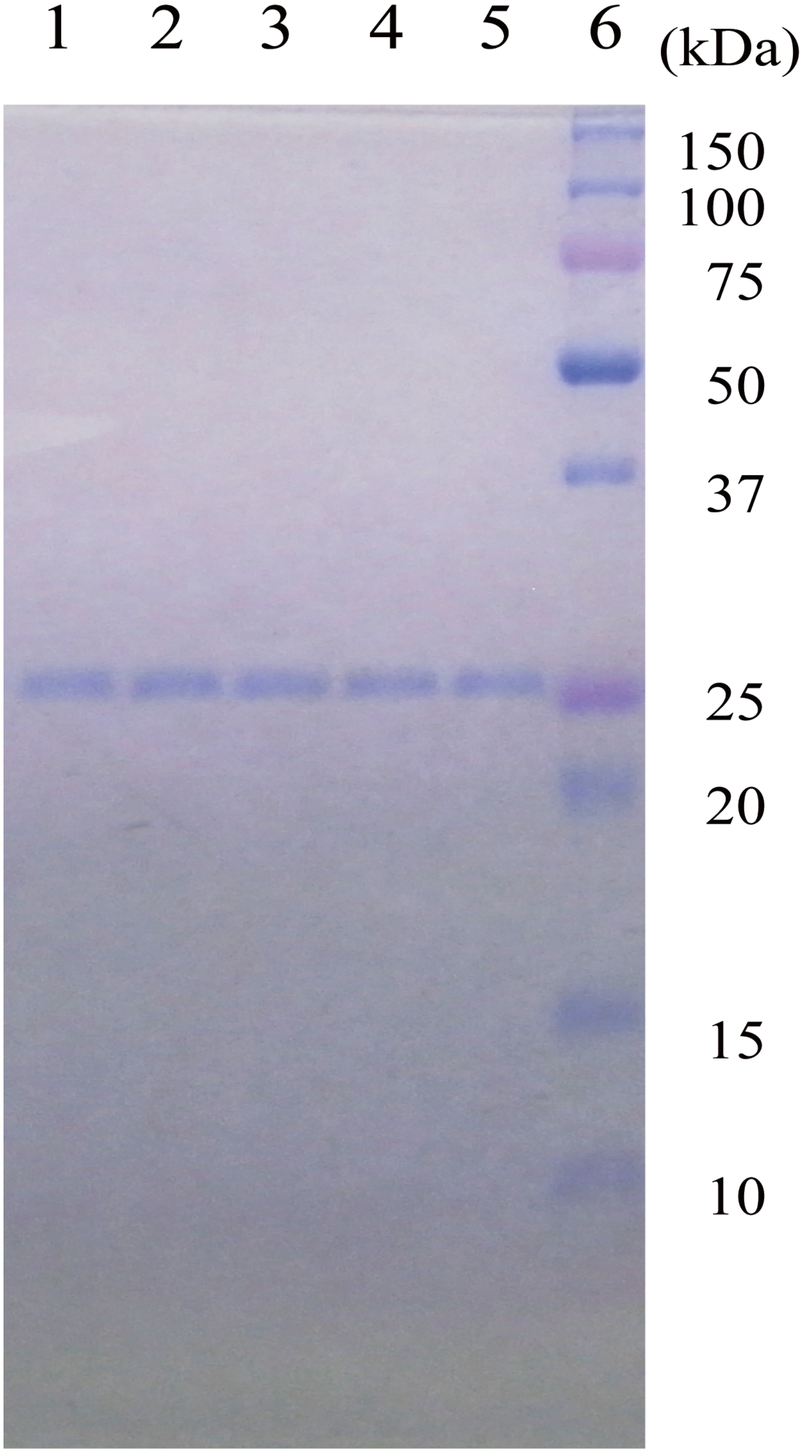
1
2
3
4
5
6
(kDa)
150
100
75
50
37
25
20
15
10

Supplementary Figure S1 Sodium dodecyl sulfate polyacrylamide gel electrophoresis (SDS-PAGE) profile of recombinant bmGSTu2 and its mutants

Purified recombinant proteins were applied to SDS-PAGE as described in the Methods section. M, protein molecular size markers; lane 1, WT; lane 2, I54A; lane 3, E66A; lane 4, S67A; lane 5, N68A.

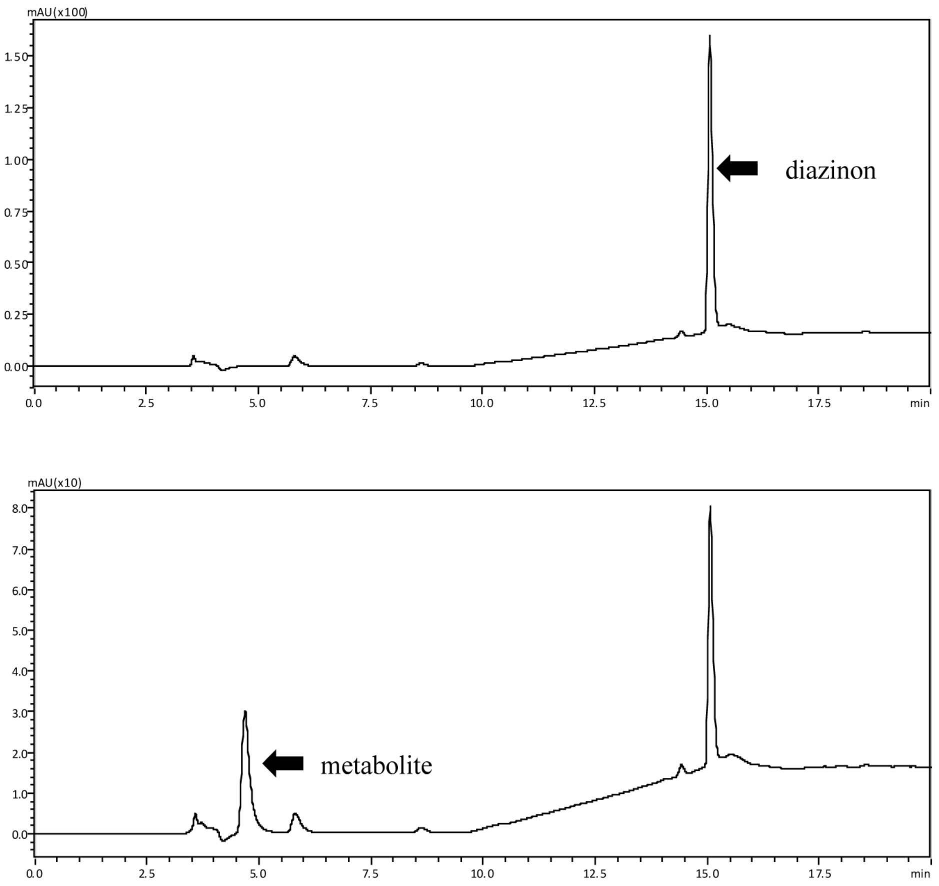

Supplementary Figure S2 Analysis of diazinon metabolites of bmGSTu2 by Liquid-chromatography (LC)

LC analysis after incubation of diazinon with bmGSTu2 and glutathione (GSH). Conditions were described in the Methods section. Diazinon and its metabolite are indicated with arrow.

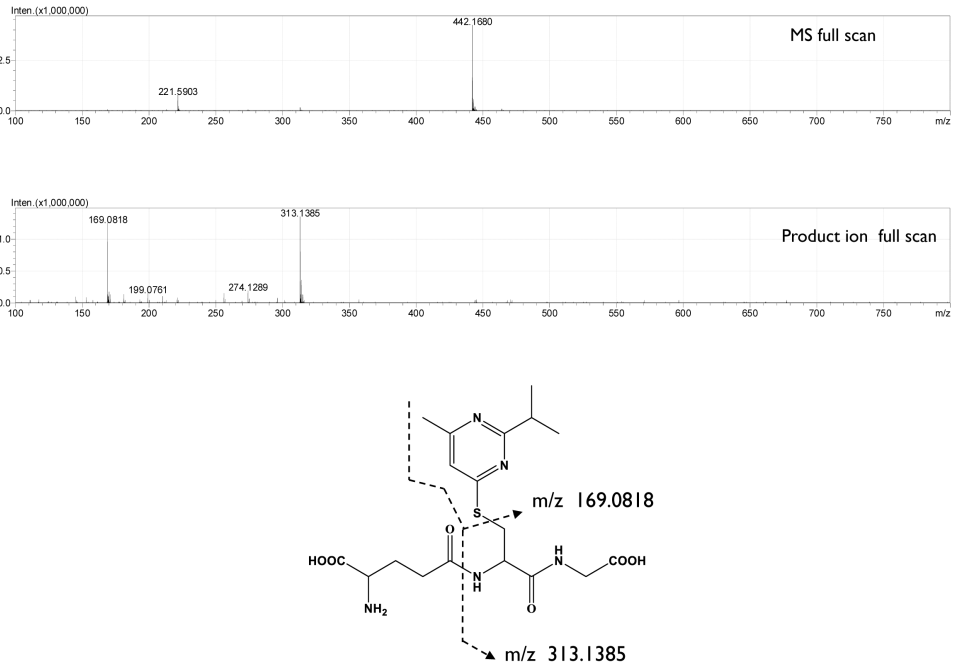

Supplementary Figure S3 Analysis of diazinon metabolites of bmGSTu2 by Mass-spectrometry (MS)/MS

MS/MS analysis of the diazinon metabolite was carried out. The ion peaks at *m*/*z* 169 and 313 correspond to the molecular ions of GSH-conjugated diazinon.

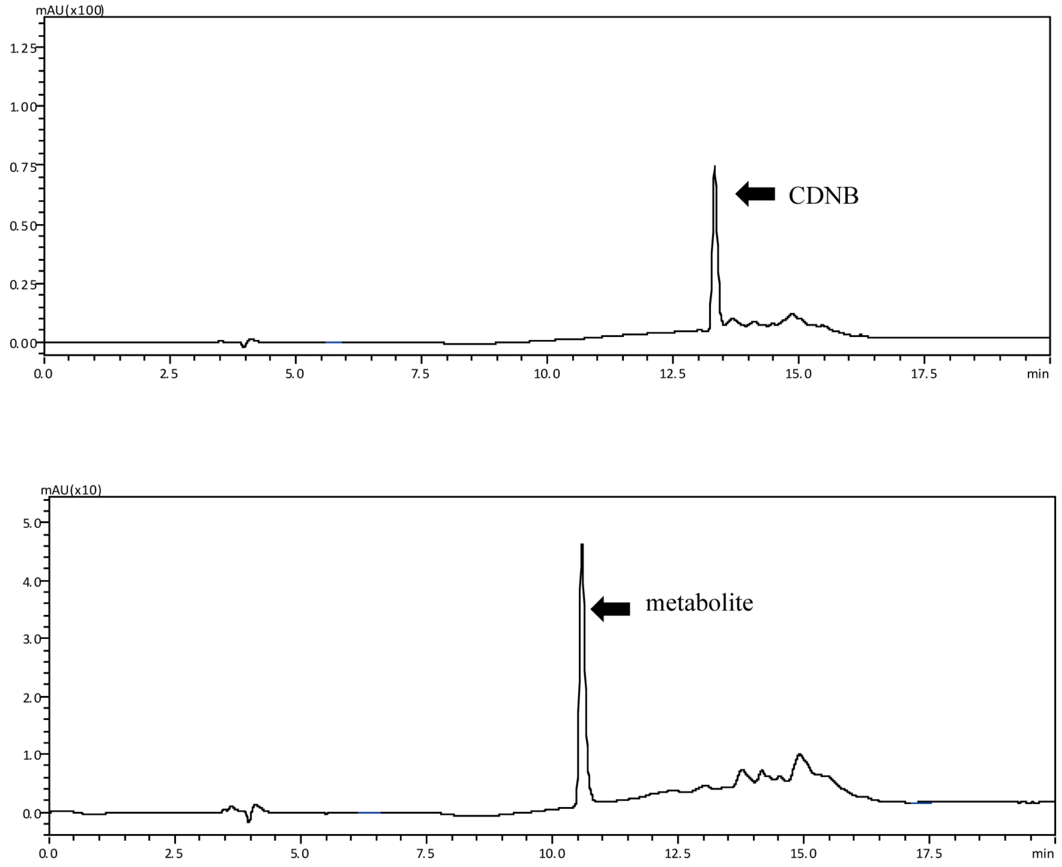

Supplementary Figure S4 Analysis of 1-chloro-2,4-dinitrobenzene (CDNB) metabolites of bmGSTu2 by LC

LC analysis after incubation of CDNB with bmGSTu2 and GSH. Conditions were described in the Methods section. CDNB and its metabolite are indicated with arrow.

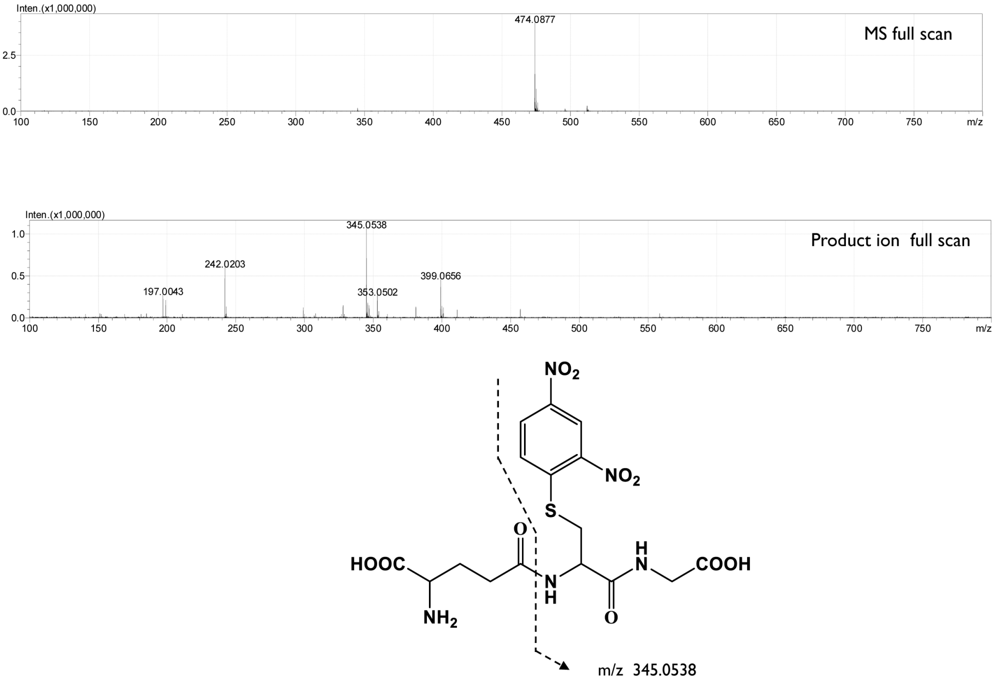

Supplementary Figure S5 Analysis of 1-chloro-2,4-dinitrobenzene (CDNB) metabolites of bmGSTu2 by MS/MS

MS/MS analysis of the CDNB conjugate was performed. The ion peaks at *m*/*z* 345 correspond to the molecular ions of GSH-conjugated CDNB.
